# Comparison of Ductile-to-Brittle Transition Behavior in Two Similar Ferritic Oxide Dispersion Strengthened Alloys

**DOI:** 10.3390/ma9080637

**Published:** 2016-07-29

**Authors:** Jesus Chao, Rosalia Rementeria, Maria Aranda, Carlos Capdevila, Jose Luis Gonzalez-Carrasco

**Affiliations:** Centro Nacional de Investigaciones Metalúrgicas (CENIM-CSIC), Avda. Gregorio del Amo 8, Madrid 28040, Spain; jchao@cenim.csic.es (J.C.); rosalia.rementeria@cenim.csic.es (R.R.); m.m.aranda@cenim.csic.es (M.A.); jlg@cenim.csic.es (J.L.G.-C.)

**Keywords:** ductile-brittle transition behavior, delamination, mechanical alloying, oxide dispersion strengthened ferritic alloy

## Abstract

The ductile-to-brittle transition (DBT) behavior of two similar Fe-Cr-Al oxide dispersion strengthened (ODS) stainless steels was analyzed following the Cottrell–Petch model. Both alloys were manufactured by mechanical alloying (MA) but by different forming routes. One was manufactured as hot rolled tube, and the other in the form of hot extruded bar. The two hot forming routes considered do not significantly influence the microstructure, but cause differences in the texture and the distribution of oxide particles. These have little influence on tensile properties; however, the DBT temperature and the upper shelf energy (USE) are significantly affected because of delamination orientation with regard to the notch plane. Whereas in hot rolled material the delaminations are parallel to the rolling surface, in the hot extruded material, they are randomly oriented because the material is transversally isotropic.

## 1. Introduction

Oxide dispersion strengthened (ODS) ferritic stainless steels are considered to be the most promising structural materials for several types of nuclear reactors, including future fusion reactors, because they combine an acceptable creep rupture strength, excellent swelling resistance, and high corrosion resistance in supercritical pressurized water [1,2,3,4]. These materials are usually produced by the mechanical alloying (MA) of yttria nano-particles and elementary powders of Fe, Cr, Al, and Ti elements [5]. The resulting powders are vulnerable to impurity contamination due to the MA processing. The consolidation of the powders is performed by hot isostatic pressing and/or hot extrusion at approximately 1000 to 1100 °C [5]. These thermomechanical processes lead to the development of a bundle-like structure of elongated grains with isolated sub-micrometric particle stringers aligned in the extrusion/rolling direction. Moreover, a strong <110> fiber texture along the extrusion/rolling direction is formed [6]. Lower fracture toughness than those of conventional ferritic stainless steels is one of the important factors limiting the use of ODS steels in the nuclear power industry. It is generally thought that the MA of ferritic steels with Y_2_O_3_ particles causes the degradation of the impact behavior of steel [7]. It was found that the mechanical alloying of the atomized tempered ferritic-martensitic steel Eurofer’97 with Y_2_O_3_ particles led to a significant degradation of the impact behavior of the steel [8]. This behavior of ODS steels is connected to the remaining porosity and morphology of Cr precipitates caused by the Hot Isostatic Pressure (HIP) consolidation process and can be partly overcome by subsequent thermo-mechanical treatment [9] or by a hot extrusion process [10]. The ductile–brittle transition behavior of ODS steels is thus influenced by both the oxide dispersion and the BCC matrix microstructure. Another issue linked to the ODS steels is their pronounced anisotropy, coming from the fabrication route of the steel by powder metallurgy and hot extrusion. The ODS steels typically contains grains elongated with a factor of 1:10 in the uniaxial direction (the extrusion direction). The plastic strain of ODS steels at fracture in the uni-axial direction is about twice that in the hoop direction, and the fracture toughness master curve in the hoop direction is shifted towards higher temperatures by about 150 °C compared to the fracture toughness master curve in the uniaxial direction [11].

This work compares and analyzes the ductile-to-brittle transition (DBT) behavior of two commercial ODS ferritic stainless steels of similar composition manufactured by two different MA routes, including hot extrusion and hot rolling.

## 2. Materials and Experimental Procedure

### 2.1. Materials

The PM 2000 alloy was provided by the Plansee Group (Reutte, Austria) in the form of an as-hot rolled tube of 100 mm diameter and 7.9 mm thickness. Material processing involved mechanical alloying and hot-compaction by extrusion, and the material was subsequently hot-rolled (~1050 °C) into a tube and air cooled to room temperature.

The MA956 alloy was provided by the Special Metal Corporation (Hereford, UK) in the form of an as-extruded bar of 60 mm diameter. The material was extruded at a temperature of 1050 °C followed by air cooling to room temperature. The chemical composition of the two alloys is listed in Table 1, as determined using X-ray fluorescence spectrometry, wet chemistry, and inert gas fusion techniques.

In general, the processing of both materials involved mechanical alloying in a shielding atmosphere of either hydrogen (PM2000) or argon (MA956), hot-compaction, hot-extrusion and/or hot rolling (~1050 °C), and air cooling. During processing, no evidence of discontinuous dynamic recrystallization involving nucleation and growth of new grains was observed, although the hot extrusion and/or hot rolling stages were conducted at very high temperatures. During tube forming, the material undergoes an extended recovery mechanism, which is characterized by a gradual increase in misorientation between neighboring subgrains that were created by recovery processes in the earlier stages of deformation [12]. The resulting dislocation substructure was a complex network of a mix of higher- and lower-angle walls characterized by misorientation angles not exceeding 2° [13]. The microstructure consists of submicrometric grains elongated in the extrusion/rolling direction [14].

### 2.2. Microstructure

The ultrafine grain size of the material was determined from secondary electron and backscattered electron images obtained in a JSM 6500F field emission gun scanning electron microscope (FEG-SEM) (JEOL, Tokyo, Japan) operating at 10 kV. Selected samples were polished to a 0.05 μm finish using a colloidal silica suspension.

**Figure 1:**
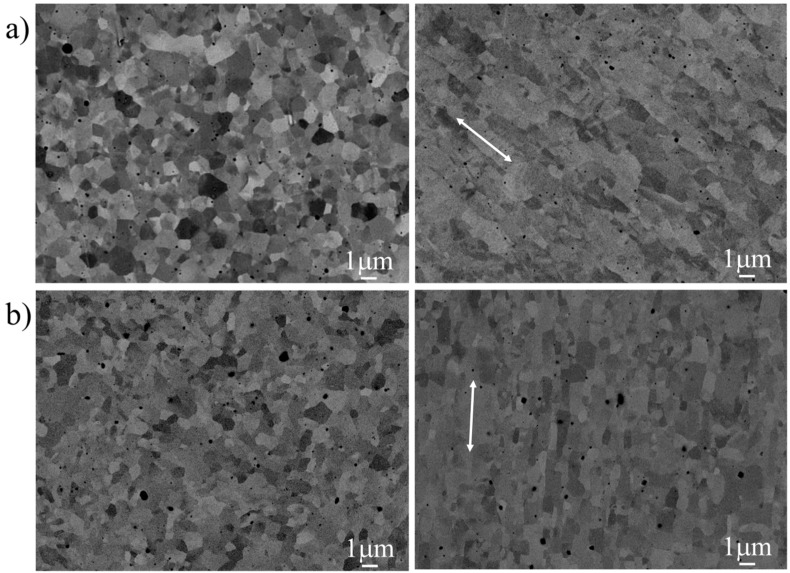
Back-scattered SEM images of the microstructure of (**a**) the transverse short (T–S) plane (**left**) and longitudinal transverse (L–T) plane (**right**) of the PM2000 alloy tube; (**b**) transverse (**left**) and longitudinal (**right**) cross sections of the MA956 alloy bar. The white arrow indicates the L direction.

Previous characterization of microstructure of the PM2000 alloy exhibited an anisotropic grain structure with equiaxed dimensions in the T–S (transverse short) plane (≈0.7 μm), but with significant elongation in the L (longitudinal) direction in the L–T (longitudinal transverse) and L–S (longitudinal short) planes (≈1.6 μm), as illustrated in Figure 1a [15]. Yttria particles with sizes ranging from 3 nm to 40 nm are mainly located at the grain boundaries [15]. Additionally, numerous large inclusions of Al_2_O_3_ (as well as Al_2_O_3_/Y-Al-O inclusions with complex compositions) were found, which preferentially formed considerably large stringers in the L–S and L–T planes; however, stringers of this inclusion type were not found in the T–S plane [15]. 

The microstructure of the MA956 alloy—similar to that observed for PM2000—presents similar equiaxed grain morphology in the transverse cross-section (≈0.7 μm), but with lower elongation (≈0.9 μm) in the extrusion direction (Figure 1b). Two main distributions of particles were observed: small particles with sizes from 5 nm to 40 nm and large particles up to 500 nm [16]. Moreover, Al_2_O_3_ particles 100 nm in size and larger and Ti(C,N) up to a few μm in size were also found [17]. Table 2 shows the microstructural features of both alloys.

### 2.3. Texture

The textures of the materials were determined by XRD in a Bruker AXS D8 (Bruker GmbH, Karlsruhe, Germany). The diffraction studies were performed with Co Kα radiation.

**Figure 2:**
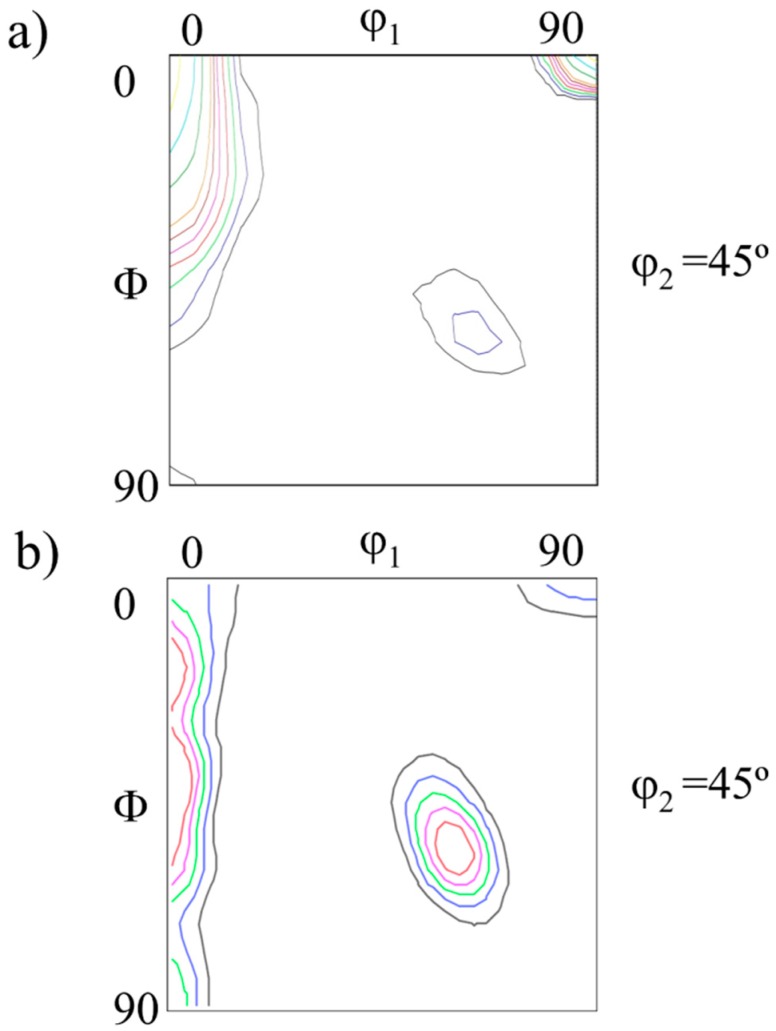
Orientation Distribution Fuction (ODF) at Φ_2_ = 45°: (**a**) PM2000 alloy; (**b**) MA956 alloy.

The texture analysis of PM2000 reveals the existence of a preferential orientation of <110> crystalline directions parallel to the rolling direction, and with a lesser intensity, to the transverse direction. Moreover, another minor intensity component exists, <100> parallel to the normal direction of the tube. The φ_2_ = 45° section of the Euler space (Figure 2a) corresponds to the T–S plane, confirming a strong incomplete α-fiber (RD‖<110>) texture with a dominant {001}<110> component— i.e., the (100) crystallographic plane parallel to the tube surface and the <110> crystallographic direction parallel to the rolling direction. 

The {110} pole in Figure 2b—which was obtained from a transverse section of the bar—shows that the MA956 alloy presents a strong α-fiber (ED‖<110>) texture. Gradients along the radial direction were only observed very close to the surface. 

### 2.4. Mechanical Testing

Tensile specimens with a gauge length of 22 mm and a diameter of 3 mm were machined from PM2000 and MA956 alloys so that the tensile axis was parallel to the longitudinal direction of the tube and bar, respectively. The specimens were tested in a range of temperatures between −196 °C and 300 °C using an initial strain rate of 5 × 10^−4^·s^−1^.

**Figure 3:**
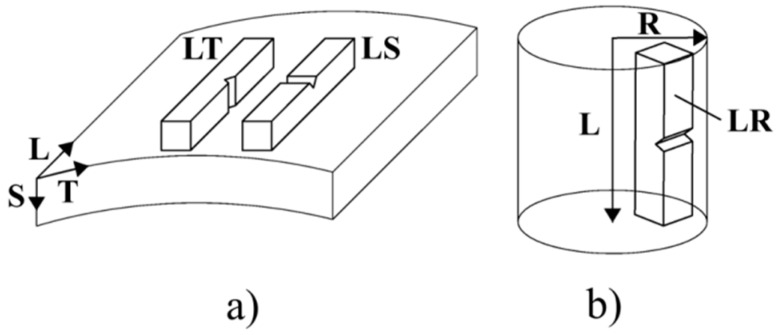
Sketch of the notch position of the Charpy specimens for (**a**) the PM2000 alloy tube and (**b**) the MA956 alloy bar.

The orientation of Charpy impact specimens was designated according to ASTM standards using a two-letter code [18]. The first letter designates the normal direction to the notch plane, and the second letter designates the direction parallel to the striking direction. In the case of the PM2000 tube, the longitudinal (L) direction is parallel to the rolling direction, the transverse (T) is parallel to the transverse direction, and the short (S) direction is parallel to the normal direction (Figure 3a). For the MA956 bar, the longitudinal L direction is parallel to the extrusion direction and the radial R direction is normal to the bar axis (Figure 3b). Sub-sized Charpy impact specimens of 55 × 7 × 4 mm^3^ with a U notch of 2 mm depth and 1 mm notch tip radius were machined in LT and LS orientations for the PM2000 alloy (Figure 3a), and in LR orientation for the MA956 alloy (Figure 3b). Charpy impact specimens were subjected to an impact energy of 300 J with an instantaneous velocity at the impact of the hammer with the specimen of 5.4 ms^−1^ in a temperature range of −197 °C to 425 °C. The ductile-to-brittle transition temperature (DBTT) was determined as the temperature corresponding to an impact energy level equal to one-half of the difference between the respective lower and upper shelf energies. A minimum of three specimens was tested at each temperature. 

## 3. Experimental Results

### 3.1. Tensile Tests

**Figure 4:**
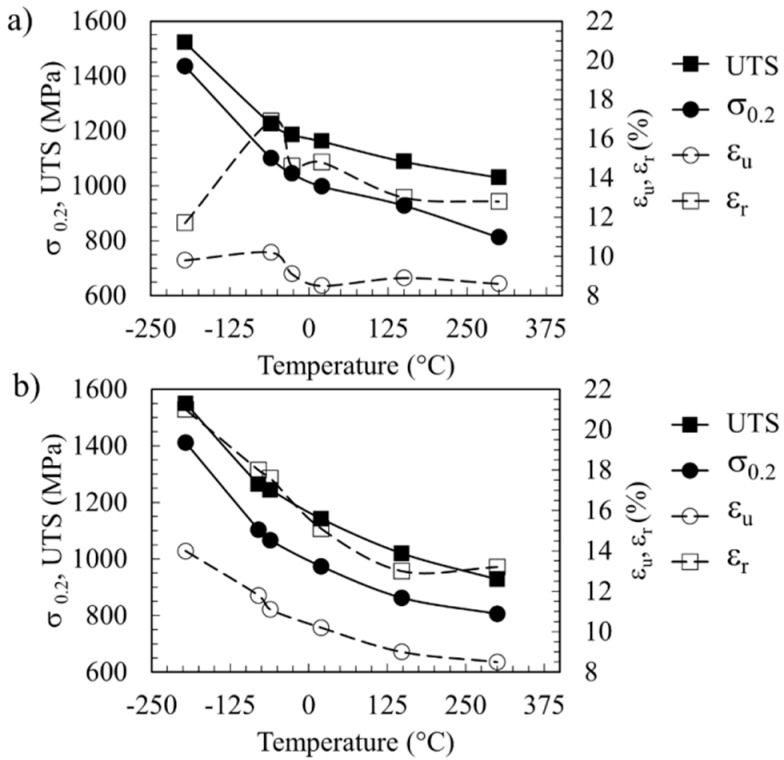
Tensile properties variation with temperature for (**a**) the PM2000 alloy and (**b**) the MA956 alloy. σ_0.2_, ultimate tensile stress (UTS), ε_u_, and ε_r_ represent 0.2% proof strength, ultimate tensile strength, uniform elongation, and total elongation, respectively.

The effect of temperature on the tensile properties of the PM2000 and MA956 alloys is shown in Figure 4a,b, respectively. The 0.2% proof stress (σ_0.2_), ultimate tensile stress (UTS) and uniform (ε_u_) and total (ε_r_, l_o_ = 5d_o_) elongations increase as the test temperature decreases for the case of MA956. By contrast, the elongation at −196 °C in PM 2000 is smaller than that obtained at higher temperatures. Strain hardening increases with increasing temperature because the difference between σ_0.2_ and UTS values increases, whereas the increase with the temperature of ε_u_ is higher than that of ε_r_.

#### Fracture Analysis of Tensile Specimens

**Figure 5:**
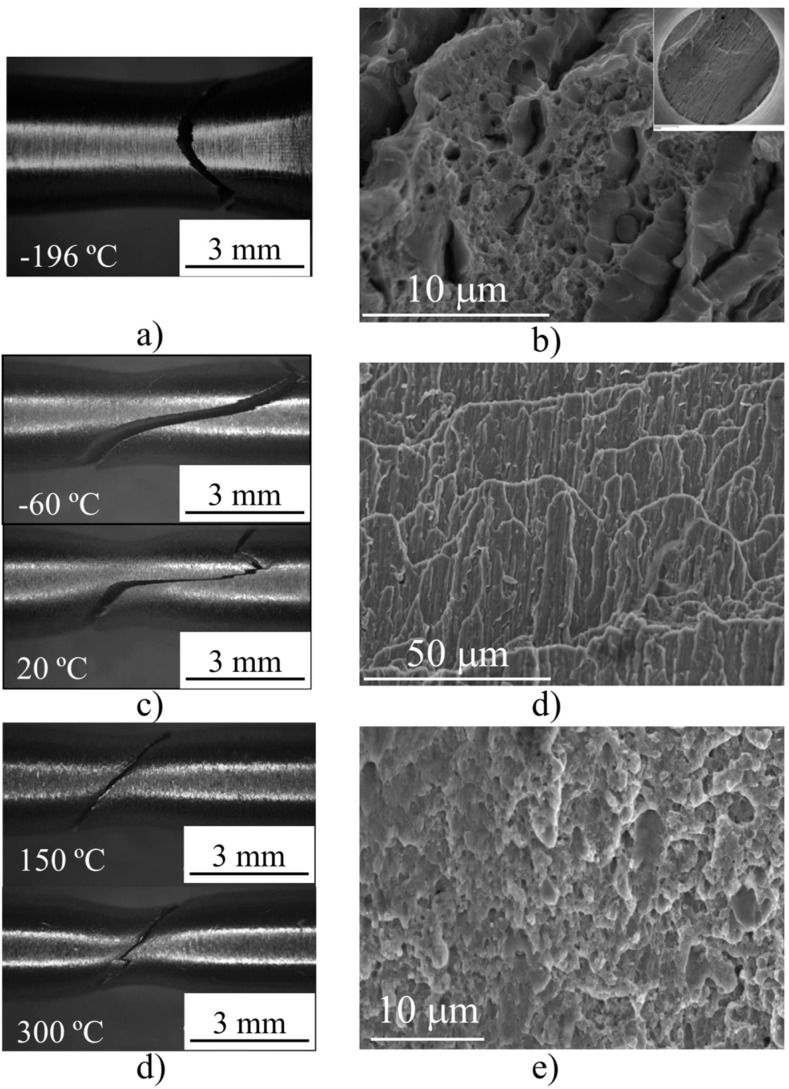
(**a**) Optical macrographs showing the breaking mode of tensile specimens of the PM2000 alloy tested at different temperatures; (**b**) SEM images showing the delaminations in the inset and the fractographic features of the fracture surface of tensile specimens tested at −196 °C; (**c**) SEM image of representative features of the fracture surface of tensile specimens tested at −60 °C and +20 °C; (**d**) SEM image of representative features of the fracture surface of tensile specimens tested at +150 °C and 300 °C.

For the PM2000 alloy, no classical transition from cleavage to ductile fracture by coalescence of microvoids was observed, but two transitions occurred: The former occurred at approximately −100 °C from the cleavage fracture along a 45° tilted plane with respect to the tensile axis, to an intergranular fracture along a 70° tilted plane with respect to the tensile axis. The second occurred between room temperature (RT) and 150°, from the intergranular fracture to the ductile shear fracture along a 45° tilted plane with respect to the tensile axis (Figure 5). It is remarkable that at −196 °C—although cleavage is the main fracture mode—incipient delaminations were also observed. For the intergranular fracture, the delaminations occurred on a plane nearly parallel to the tube surface—i.e., to the L–T plane. For the entire range of studied temperatures, necking preceded the fracture (Figure 5).

**Figure 6:**
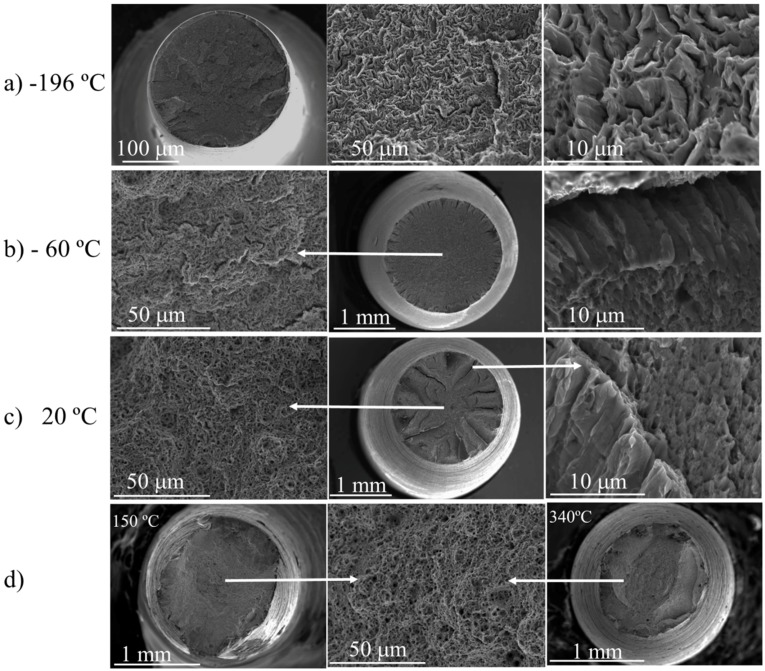
(**a**) SEM images in progressively higher magnifications of the fracture surface of tensile specimens tested at −196 °C; (**b**) SEM images of the fracture surface of tensile specimens tested at −60 °C, showing the fractographic features at the center of the cross section (**left**) and at the edge near the delaminations (**right**); (**c**) SEM images of the fracture surface of tensile specimens tested at +20 °C, showing the fractographic features at the center of the cross section (**left**) and at the edge near the delaminations (**right**); (**d**) SEM images of the fracture surface of tensile specimens tested at +150 °C (**left**), 340 °C (**right**), and representative fractographic features at high magnification (**middle**).

Unlike the PM2000 alloy, a classical transition was observed for the MA956 alloy from the cleavage fracture (Figure 6a) to the ductile fracture (Figure 6b) as temperature increased. However, some interesting fractographic features must be highlighted. At −60 °C, evenly-distributed radial delaminations of approximately 150 µm depth were observed on the surface (Figure 6b, **left**). The fractographic features of the delamination surfaces (Figure 6b **right**) are similar to those found for the PM2000 alloy (Figure 5c). At RT, the number of delaminations decreased, but the depth increased until values were in excess of 500 μm, as illustrated in Figure 6c. At +150 °C and 340 °C, no delaminations were observed (Figure 6d **left** and right, respectively).

### 3.2. Charpy Impact Tests

**Figure 7:**
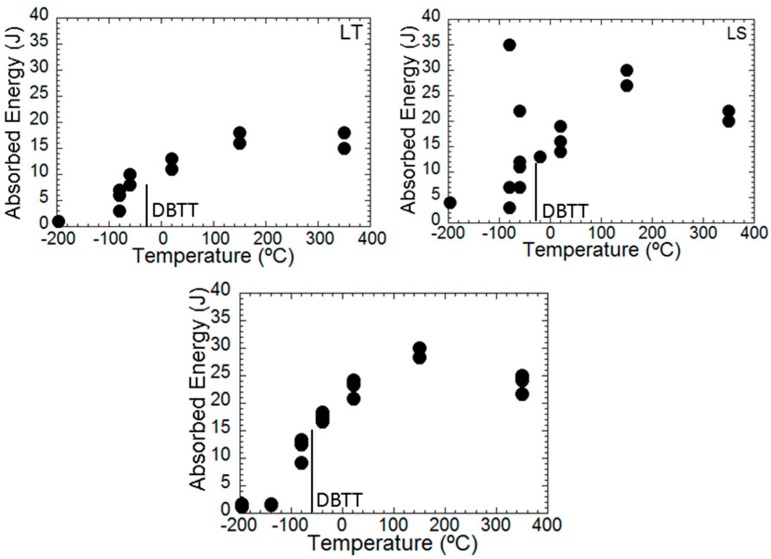
Variation with temperature of the U-notch Charpy absorbed energy for (**a**) LT-notched specimens of the PM2000 alloy; (**b**) LS-notched specimens of the PM2000 alloy; and (**c**) the MA956 alloy. DBTT: ductile-to-brittle transition temperature.

The DBTT curves of the PM2000 alloys for LT and LS notch positions are presented in Figure 7a,b, respectively, whereas that of the MA956 alloy is shown in Figure 7c. A trend for the Charpy impact values is observed in this figure. The absorbed energy values increase in a sigmoidal shape as the temperature increases from a lower shelf (LSE) in low temperatures to an upper shelf (USE) in high temperatures, which has previously been reported in other ferritic steels. In the case of the LS notch position of the PM2000 alloy and for the MA956 alloy, no USE was observed, but a maximum value of absorbed energy at approximately 150 °C was recorded. The DBTT temperature was determined from the USE value obtained at 350 °C. Moreover, in the case of the LS notch orientation, a great dispersion in the absorbed energy value was observed at −60 °C. 

#### 3.2.1. Fracture Analysis of Tested Charpy Impact Specimens

##### LT Specimens of PM2000 Alloy 

**Figure 8:**
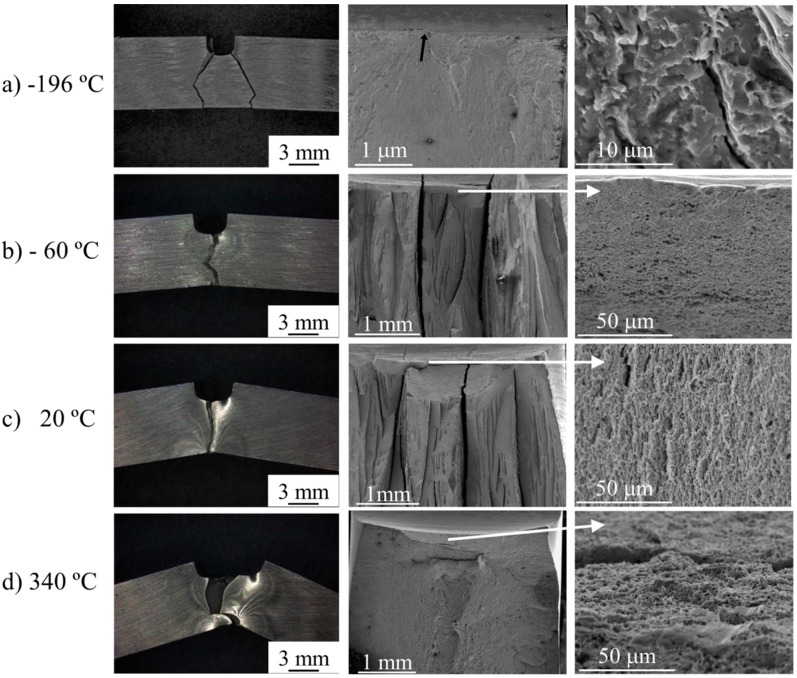
Appearances of LT-notched Charpy impact test specimens of the PM2000 alloy after fracturing at (**a**) −196 °C; showing the fracture profile (left), the fracture initiation point on the fracture surface (**middle**), and the microscopic features at high magnification (**right**); (**b**) −60 °C; showing the fracture profile (**left**), the delamination on the fracture surface (**middle**), and the microscopic features of the fracture between delaminations (**right**); (**c**) +20 °C; showing the fracture profile (**left**), the delaminations on the fracture surface (**middle**), and the microscopic features of the fracture between delaminations (**right**); (**d**) 340 °C; showing the fracture profile (**left**), the ductile aspect of the fracture surface with a delamination parallel to the notch basis (**middle**), and the microscopic features between delaminations (**right**).

For temperatures ranging from −196 °C to −80 °C, the fracture presents a zigzag appearance on planes inclined ≈30° with respect to the notch plane (Figure 8a). In this temperature range, no delaminations were found on the fracture surface. In test temperatures between −30 °C and 220 °C, the fracture presents a ductile aspect with delaminations parallel to the tube surface (Figure 8b **middle** and Figure 8c **middle**). The number of delaminations decreased as the temperature increased. The fracture mechanism associated with the delaminations is of an intergranular-brittle type.

##### LS specimens of PM2000 Alloy

**Figure 9:**
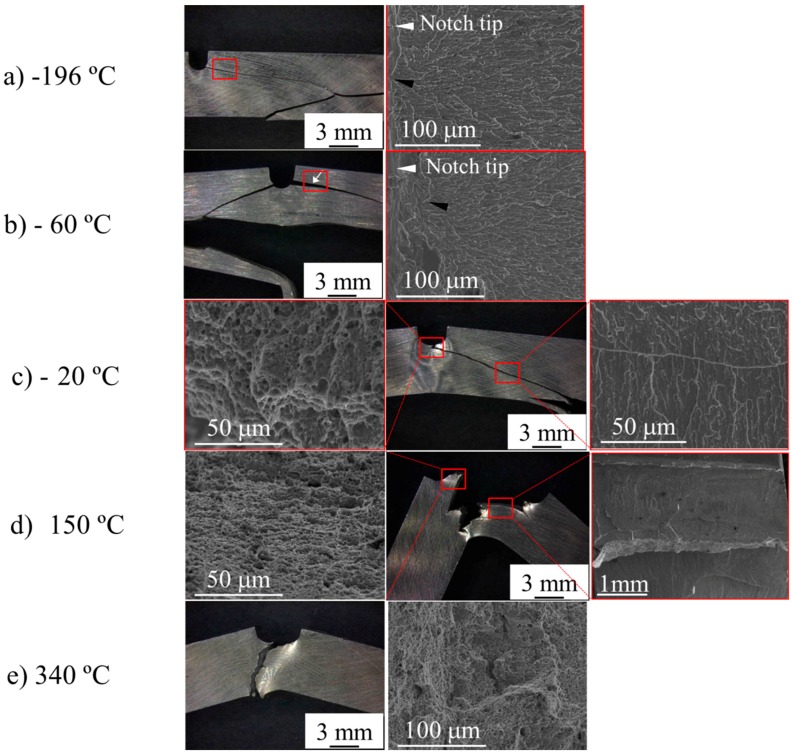
Appearances of LS-notched Charpy impact test specimens of the PM2000 alloy after fracture at (**a**) −196 °C; showing the fracture profile (**left**) and fracture initiation point on the fracture surface (**right**); (**b**) −60 °C; showing the fracture profile (**left**) and fracture initiation point on the fracture surface (**right**); (**c**) −20 °C; showing the fracture profile (**middle**), the microscopic features of stable ductile tear (**left**), and microscopic features of the fracture surface on the 70°-inclined plane; (**d**) +150 °C; showing the fracture profile (**middle**), the microscopic features of stable ductile tear (**left**) and microscopic features of the fracture surface on the 70°-inclined plane (**right**); (**e**) 340 °C; showing the fracture profile (**left**) and the microscopic features of the fracture surface (**right**).

Despite the differences in absorbed energy, a similar fracture behavior was observed for specimens tested at temperatures between −196 °C and RT (Figure 9a–c). In general, the specimens fractured along the ≈70° tilted planes to the notch plane present a brittle appearance (Figure 8a,b **left** and Figure 8c **middle**). An in-depth analysis of the fracture surface reveals a mixture of intergranular facets with ductile steps between them. The large differences in absorbed energy for a given testing temperature could be attributed to the “crack arrester” [19] effect of the delaminations, and to the successive positions of the delaminations with respect to the instantaneous crack tip. At temperatures above RT, the fracture presents a ductile aspect with isolated delaminations parallel to the tube surface.

##### MA956 Alloy

**Figure 10:**
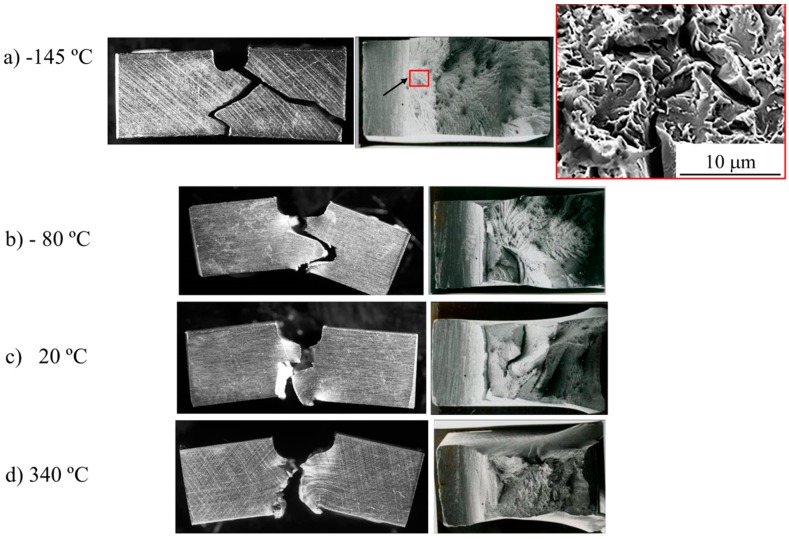
Appearance of Charpy impact test specimens of MA956 alloy after fracture at (**a**) −145 °C; showing the fracture profile (**left**), the location of the fracture initiation (**middle**), and the microscopic features of the fracture surface (**right**); (**b**) −80 °C; showing the fracture profile (**left**) and the location of the fracture initiation point after of ductile stable crack growth and the delaminations (**right**); (**c**) +20 °C; showing the fracture profile (**left**) and the fracture surface in which delaminations are observed; (**d**) 340 °C; showing the fracture profile (**left**) and the ductile aspect of the fracture surface (**right**).

From −196 °C to −80 °C, the fracture presents a zigzag morphology on planes inclined approximately ≈60° to the notch plane. At −196 °C and −145 °C, the fracture initiates at the notch tip, but no microstructural features can be detected following the radial lines in progressively higher magnifications (Figure 10a). Irregular delaminations or secondary cracks were also observed (Figure 10b,c). At −80 °C, a cleavage fracture occurred after a small amount of stable crack growth by a ductile mechanism (Figure 10b). The fracture initiated ahead of the tip of the ductile crack at the intersection of the delaminations with the main fracture, and occasionally at Ti(C, N) particles. SEM analysis of the fractographic features of the fracture surface of the delaminations revealed that the fracture was of an intergranular-brittle type. At higher temperatures, the fracture occurred by a ductile mechanism with the presence of isolated delaminations, except at 340 °C (Figure 10d), where delaminations were not observed.

## 4. Discussion

The primary purpose of this study is to analyze the differences in LSE, DBTT, and USE values of DBT curves of PM2000 and MA956 alloys, considering the differences in manufacturing routes—which cause differences in chemical composition, texture, and as-received microstructure, as highlighted in previous sections.

Figure 7a,b and Table 3 show that the LSE value for LS-notched specimens is significantly higher (3 J) than that of 1 J for LT-notched specimens. This can in all likelihood be attributed to the differences in the failure mechanics. Thus, in LS-notched specimens, the fracture occurred along a 70°-tilted plane with respect to the notch plane by an intergranular mechanism, whereas in the LT-notch specimen, a fracture occurred along a ≈30°-tilted plane with respect to the notch plane by a cleavage mechanism. Actually, the LSE-values for the LS-notch and LT-notch specimens could not be compared, because the fracture mechanism associated with such values are different. Despite this finding, LS-notched specimens indicate that there must be a contribution to the absorbed energy in the Charpy impact from the bending of the un-notched ligament of the specimen, simultaneous to the unsteady progress of the fracture along a 70°-tilted plane with respect to the notch plane.

The LSE value of the MA956 alloy is between the LSE values of the PM2000 alloy for the LS-notch and LT-notch specimens (Table 3). It is remarkable that the LSE value of MA956 is 50% higher than that of the LT-notch specimen of the PM2000 alloy, even though the effective grain size and fracture mechanism (cleavage) are similar in both cases. The only difference lies in the presence of a higher density of small secondary cracks or delaminations in MA956 than in PM2000. These delaminations could delay the quick spread of the cleavage fracture and then increase the absorbed energy during fracturing in the Charpy test. The authors are aware that the analysis of LSE-values must be performed very carefully for two reasons. First, the accuracy of the scale gradation of the Charpy tests machine was 1 J. Second, although the temperature of the bath in which the specimens were immersed was carefully controlled, the time between specimen removal from the bath and fracturing might vary from specimen to specimen or slightly exceed the five seconds allowed for transferring and fracturing of the specimens. For these reasons, the authors cannot positively infer anything about LSE-values of the MA956 and PM2000 alloys in terms of LT-notch position.

It is generally accepted that the rising part of the DBT-curve is the transition region between fully-cleavage and fully-ductile fracture processes, which is associated with a mixture of both cleavage and ductile fracture mechanisms [20,21,22]. It is very common to relate the rise of Charpy impact energy to an increase in the proportion of ductile fracturing. Moreover, when a large number of nominally identical specimens are tested at the same temperature in the DBT region, the resulting values present greater scatter than those in the LSE and USE regions. The increase in the scatter is linked to the variability in the proportions of brittle and ductile fractures from specimen to specimen. In general, the results in Figure 7a (PM2000 LT-notch position) and Figure 7c (MA956) confirm the above trends of the DBT curves. However the low scatter observed in the absorbed energy at all temperatures of the DBT is remarkable. Moreover, the scatter observed in the DBT region is similar to that observed in the USE region, but curiously, it is higher than that observed in the LSE region. 

The situation in the LS-notched specimens is completely different from that shown for the LT-notched specimens (Figure 7a,b). Figure 7b shows enormous scatter in the values of absorbed energy at −80 °C and −60 °C in LS-notched specimens, which is due to microstructure morphology and the texture of the material [15]. Moreover, it has been recently reported that the PM2000 alloy displays similar behavior to that of a laminated material [23].

The analysis of Cottrell–Petch [24,25] provides a useful basis for discussion of the results in the transition region of the DBT curve. In the Cottrell–Petch model, the ductile-to-brittle transition occurred when

(1) $$k_{y}^{2}+\sigma_{o}k_{y}d^{1/2}=\sigma_{y}k_{y}d^{1/2}\;\geq C\mu \gamma$$

where k_y_ is the Hall–Petch slope, σ_o_ is the lattice friction stress, d is the grain size, σ_y_ is the flow stress, C is a constant, μ is the shear modulus, and γ is the effective surface energy.

Because there are only slight differences in the effective grain size and 0.2% flow strength, we considered the texture and the variations in the chemical composition through its possible effects on k_y_ and γ as the factors determining the DBTT and USE. 

### 4.1. Chemical Composition

In a ferritic matrix, Al inhibits the cross slip (which produces an increase in the strength), and in accordance with results of Figure 7a,c, an increase in the DBTT and a decrease in the USE [26,27]. However, these effects are unrelated, because the flow strength of both materials is not substantially different.

The strong effect of (C + N) on the toughness of ferritic stainless steels has long been known [26,28]. When the concentration of (C + N) interstitials is higher than 0.015%, an increase in C and/or N causes an increase in the DBTT and a decrease in the USE [26,28]. The physical reason behind these results is the very low solid solution of C and N in the ferritic matrix, which causes the precipitation of chromium carbides and nitrides, mainly in grain boundaries. Accordingly, because the (C + N) content of the MA956 alloy is considerably higher (0.032%) than that of the PM2000 alloy (0.013%), the following can be expected: DBTT_(MA956)_ > DBTT_(PM2000)_ and USE_(MA956)_ < USE_(PM2000)_, which is in contrast with the results of Figure 7a,c. Titanium additions of up 0.66% to ferritic stainless steels of similar Cr-composition tended to lower the DBTT and increase the USE compared with steels of similar (C + N) content containing no Ti [22]. The embrittlement effect of the (C + N) content is considerably reduced by the Ti-addition, due to the preferred tendency of Ti to recombine with C and N for precipitate as Ti(C,N) with Cr precipitates as Cr(C,N). In fact, no addition of Ti would be necessary for the PM2000 alloy because the (C + N) content is already too low to cause embrittlement of the matrix. In any case, the volume fraction of Ti(C,N) precipitates in the MA956 alloy should be significantly higher than in the PM2000 alloy, and thus the following can be expected: DBTT_(MA956)_ > DBTT_(PM2000)_ and USE_(MA956)_ < USE_(PM2000)_, which, as above, would be in contrast with the results of Figure 7a,c.

### 4.2. Texture

Previous works have demonstrated that BCC materials with <110> fiber texture present a natural strong tendency for delaminations along (100) planes when the material is stressed in the <110> direction [29]. The reason for this behavior is due to the very high texture-induced internal tensile stresses, which were produced by the propensity of the material with this texture to deform in plane-strain conditions [29,30]. The effect of the delaminations on the DBT behavior depends on the relative orientation of delaminations and the notch plane. Thus, for LT-notched specimens of the PM2000 alloy—in which the fracture advances in the “crack divider” mode—the delaminations cause a decrease in both the DBTT and USE with respect to those of monolithic material [15]. However, for LS-notched specimens—in which the fracture advances in the “crack arrester” mode—the delaminations cause a decrease in the DBTT and an increase in the USE, although a convergence of the USE was noted at high temperatures [15]. Regarding the PM2000 and MA956 alloys, an important difference in the texture must be noted. The MA956 alloy is transversely isotropic because axisymmetric forms during the extrusion processing, whereas in the PM2000 alloy, the tangential and radial directions become parallel to the <110> and <100> directions during the rolling processing. From the comparison of the DBT behavior of the MA956 and PM2000 alloys, it seems that the transverse isotropy could play a positive role in the absorbed energy in the Charpy impact test. This useful effect could be attributed to the random orientation and discontinuous nature of the delaminations.

## 5. Conclusions

The discussion of the results following the analysis of Cottrell–Petch allows us to reach the following conclusions on the differences in the DBTT between the MA956 and PM2000 alloys studied:

1. Hot-extrusion and hot-rolling manufacturing routes do not induce significant differences in the as-received microstructure, but do cause slight changes in the homogeneity of the texture and oxide distribution. Such differences do not indicate meaningful changes in the tensile properties of both MA 956 and PM 2000 ODS ferritic stainless steels, but significant changes in the DBTT and USE.
2. Despite the higher (C + N) content of the MA956 alloy (0.032%) than that of the PM2000 alloy (0.013%), which should cause DBTT_(MA956)_ > DBTT_(PM2000)_ and USE_(MA956)_ < USE_(PM2000)_, the opposite tendency is observed.
3. This apparent contradiction could be explained on the basis of the particular texture in the as-received conditions of the two alloys. The MA956 alloy is transversely isotropic because of axisymmetric forming during the extrusion processing, whereas in the PM2000 alloy during the rolling processing, the tangential and radial directions become parallel to the <110> and <100> directions, respectively, with a strong {001}<110> component. That is, the (100) crystallographic plane parallel to the tube surface. Therefore, for LT notched specimens of the PM2000 alloy—in which the fracture advances in the “crack divider” mode—the delaminations cause a decrease in both the DBTT and USE with respect to those of monolithic material. However, for LS-notched specimens—in which the fracture advances in the “crack arrester” mode—the delaminations cause a decrease in the DBTT and an increase in the USE, although a convergence of the USE was observed at high temperatures.

## Figures and Tables

**Table 1:** Chemical composition of the PM2000 and MA956 alloys.

Material	Chemical Composition (wt. %)							
	C	Si	Mn	P	S	Cr	Al	Ti
**PM2000**	<0.01	0.095	0.037	<0.02	<0.005	18.60 ± 0.08	5.2 ± 0.1	0.54
**MA956**	<0.012	0.12	0.097	<0.02	0.007	19.33 ± 0.08	4.7 ± 0.1	0.44
**Material**	**Chemical Composition (wt. %)**							
	Co	Ni	Cu	O	Y	N	Y_2_O_3_^(*)^	
**PM2000**	0.039	0.03	0.015	0.091 ± 0.02	0.391		0.5	
**MA956**	0.066	0.101	0.021	0.108 ± 0.015	0.376	0.0200 ± 0.0015	0.5	

(*) Nominal composition.

**Table 2:** Geometric measures of the microstructural characteristics and texture of the PM2000 and MA956 alloys.

	Grains			Dispersoids			
Material	D_eff_ (T–S) (μm)	l (LS) (μm)	GAR	Size nm	λ_avg_ nm	f_vol_ %	Texture
**PM2000**	0.7	1.6	2.3	19	121	1.1	(100) [100]
**MA956**	0.7	0.9	1.4	16 [8]	180 [8]	1.3 [8]	α-fiber <110>‖ED

D_eff_—effective diameter; l—length; GAR—grain aspect ratio; λ_avg_—distance between dispersoids; f_vol_—volume fraction.

**Table 3:** Values of the parameters of the DBT curves.

Material	LSE (J)	DBTT (°C)	USE (J)
PM2000-LT	1.0	−30	16.5
PM200-LS	3.0	−30	28 (*)
MA956	1.5	−60	27 (*)

LSE—Lower Shelf Energy; USE—Upper Shelf Energy; DBTT—Ductile-to-Brittle Transition Temperature. (*) Maximum value instead USE.
